# Stewardship in your pocket: launching Colorado’s free antimicrobial mobile application

**DOI:** 10.1017/ash.2026.10418

**Published:** 2026-06-01

**Authors:** Joana Dimo, Sarah K. Parker, Matthew J. Weber, Matthew A. Miller, Kira E. Frappa, Christine E. MacBrayne, Katherine C. Shihadeh, Margeret M. Cooper, Lauren R. Biehle, Timothy C. Jenkins, Michael J. Bozzella, Leigh Anne Bakel

**Affiliations:** 1 Pediatric Infectious Diseases, University of Colorado School of Medicine: University of Colorado Anschutz Medic, USA; 2 Pharmacy, University of Colorado School of Medicine: University of Colorado Anschutz Medic, USA; 3 Pharmacy, Denver Health Medical Center: Denver Health Main Campus, USA; 4 Colorado Department of Public Health and Environment, USA; 5 Denver Health Medical Center: Denver Health Main Campus, USA; 6 Hospital Medicine, University of Colorado School of Medicine: University of Colorado Anschutz Medic, USA

## Abstract

**Objective:**

The aim was to describe the development and implementation of Firstline (www.Firstline.org), a mobile antimicrobial stewardship (ASP) application (app) created to address barriers to robust ASP in Colorado hospitals.

**Design:**

A mixed-methods pragmatic implementation study using the RE-AIM (Reach, Effectiveness, Adoption, Implementation, Maintenance) framework was conducted from October 2023–December 2025.

**Methods:**

ASP teams at Children’s Hospital Colorado and Denver Health partnered with Firstline to develop the app using locally curated content. The app launched in October 2023 with a multifaceted dissemination campaign. Reach was assessed using geocoded user data. Effectiveness and adoption were evaluated using cross-sectional surveys at two time points. Implementation was characterized by analyzing utilization patterns. Maintenance was assessed by tracking monthly active users over two years.

**Results:**

Within the first year, 4,409 unique Colorado users downloaded the app, representing 15% of state clinicians (19% in Denver Metro; 5–17% in rural regions). Survey respondents (n = 215) reported high satisfaction: 63.9% and 72.9% found the app very easy to use, 71.3% and 83.2% reported enhanced antibiotic selection knowledge, and 100% advocated for ongoing support. Among respondents, 10% reported their facilities had formally adopted Firstline guidelines. The most frequently accessed content included common, high-impact infections. Monthly active users remained stable (average 871) over two years.

**Conclusions:**

We demonstrated use of Firstline across Colorado, with increasing engagement in rural areas. High user satisfaction, sustained engagement, and emerging institutional adoption suggest this “stewardship in your pocket” model offers a unique, scalable approach to extending ASP support beyond academic medical centers.

## Introduction

Inappropriate antimicrobial use is a key driver of antimicrobial resistance.^1,2^ Inappropriate antibiotics increase the risk of resistant bacterial infections, inflammatory and autoimmune conditions, obesity, adverse drug events, and overall increased healthcare costs.^3–11^

Antimicrobial Stewardship Programs (ASPs) are responsible for coordinating efforts to monitor and facilitate appropriate antibiotic use.^12,13^ ASPs are associated with substantial benefits including reduced rates of resistant infections and adverse drug events, improved patient outcomes, and significant cost savings.^14–18^ Robust ASPs are often limited to large academic medical centers with infectious diseases (ID) specialists and ID-trained pharmacists. Colorado designates 47 of its 64 counties as rural or frontier, defined by no metropolitan areas over 50,000 residents or fewer than 6 people per square mile.^19^ Of the state’s 88 hospitals, 43 operate in rural or frontier counties, and 17 counties lack a hospital entirely.^20,21^ While most Colorado hospitals have ASPs to meet reimbursement and accreditation requirements, many lack the resources and expertise to develop local guidelines, antibiograms, and infection control protocols.^22–27^ Similarly, the need for pediatric-specific guidelines and antibiotic dosing resources was identified as a barrier to robust pediatric stewardship among Colorado hospitals.^28^

To address the identified barriers to well-rounded ASPs among Colorado hospitals, Children’s Hospital Colorado (Children’s Colorado) developed an ongoing partnership with the Colorado Department of Public Health and Environment (CDPHE).^29^ One objective of this partnership is to provide stewardship-focused guidance to Colorado hospitals. As a method of accomplishing this, Children’s Colorado, in partnership with Denver Health, contracted with Firstline (www.firstline.org) to develop and implement a mobile ASP application (app), which launched in October 2023, to improve access to ASP resources across Colorado.^30^ Firstline provides pediatric and adult guidance through separate sites for Children’s Colorado and Denver Health, respectively, and is completely free to use (mobile or online).^30^ Mobile apps have the potential to support ASP implementation by promoting guideline-concordant antibiotic prescribing and information dissemination, though data on their effectiveness is variable.^31–34^

The specific aim of this pragmatic study was to describe the development and implementation of the Children’s Colorado and Denver Health Firstline mobile apps across Colorado using the RE-AIM framework by: (1) **Reach:** Assessing the proportion of clinicians reached, demographics, and geographic distribution of Firstline users, (2) **Effectiveness:** Evaluating user-reported satisfaction and perceived impact on prescribing behavior, (3) **Adoption:** Describing the characteristics of the institutions and teams involved in app development and assessing uptake of Firstline guidelines by external facilities; (4) **Implementation:** Documenting the process of app deployment, dissemination, and integration into clinical workflows and characterizing utilization patterns; and (5) **Maintenance:** Exploring use patterns and content updates over time.

## Methods

This is a mixed-methods pragmatic implementation study using the RE-AIM framework conducted from October 2023 to December 2025.

### Reach

#### Practitioner data

Practitioner data was collected through two databases. Children’s Colorado Provider Relations provided data from Definitive Healthcare. This was combined with data from the Department of Regulatory Agencies Professional and Occupational Licenses Information Marketplace, with duplicates deleted, to create a comprehensive list. The list was narrowed to physicians and advanced practice providers (APPs) to attempt to isolate clinicians that prescribe medications and pharmacists, as they compromise a high number of Firstline users in the numerator. No metric was available to identify those who specifically prescribe antibiotics. The resulting clinician list represented the denominator for the statewide rate of Firstline use.

The total number of Firstline users, defined as individuals who downloaded and used the Children’s Colorado or Denver Health app at least once, was established using internet protocol address geocoding, provided by the Firstline analytic team. Due to a Firstline system update, this data was collected only from October 2023 to September 2024 and was not comparable afterwards. This data represented the numerator for Firstline use out of all practitioners. Of note, the numerator includes all clinicians (physicians, pharmacists, nurses, etc.) as total users could not be further refined accurately by clinician type. From launch through December 31, 2025, the number of sessions, defined as a window of Firstline use within a 30-min period, by geocoded location was also collected to further assess overall app engagement.

#### Firstline utilization metrics

At initial log-in, users respond to three questions regarding their location (region of the state), clinical role (eg, physician, APP) and practice setting (eg, inpatient, emergency department). These de-identified data were linked to app engagement (ie, click data), allowing analysis of use by user characteristics.

### Effectiveness

The team designed a Research Electronic Data Capture (REDCap) survey to assess Firstline user satisfaction and distributed it at two time points: April 2024 and February 2025. The survey contained 18 questions including demographics, app utilization, app satisfaction, perceived change in practice or knowledge, and feedback (Supplemental File 1). The survey link was sent via push notification to all users of the Children’s Colorado and Denver Health Firstline apps. CDPHE distributed the survey through the Health Facilities and Emergency Medical Services Division messaging portal and a statewide newsletter and sent it to hospital ASP contacts. Children’s Colorado hosted the REDCap tools used to collect and manage the data.

### Adoption

#### Antimicrobial stewardship team

This was a collaborative effort among ASP teams at Children’s Colorado and Denver Health. Children’s Colorado is a large, free-standing children’s hospital with 632 beds across four campuses that provides support and specialty resources to a surrounding seven-state area. Denver Health is a comprehensive academic health center and Denver’s primary safety net hospital with 525 mostly adult beds and a level 1 trauma center. The Children’s Colorado team that developed the app content was comprised of the medical director of ASP (SKP), three ID pharmacists (MAM, KEF, CEM), two ID physicians (MJB, SKP), one hospitalist (LAB) who is also the medical director of the clinical pathways program, one ID fellow (JD), and one research services professional (MJW). The team at Denver Health included the medical director of ASP (TCJ) and two ID pharmacists (MMC, KCS); they adapted pathogen content from the Children’s Colorado version and otherwise built their own content.

#### Development of the Firstline app intervention

The team contracted with Firstline (www.Firstline.org), a third-party developer, to build the app. Firstline was selected for its proven experience in developing and disseminating ASP content, user-friendly interface, ability to track user activity and characteristics, and its capacity to make content publicly accessible at no cost to users. Additionally, Firstline includes features desirable for rapid dissemination of new guidance and important alerts, such as push notifications, and collects data on app use amenable to iterative improvement.

Pediatric and adult content is featured on the Children’s Colorado and Denver Health apps, respectively. Content includes (1) facility-specific ID guidelines, (2) pathogen information, (3) antimicrobial information, and (4) infection prevention, ASP, and national resources. Children’s Colorado and Denver Health existing clinical pathways, which are publicly available, were adapted for app format.^35,36^ Pathogen information includes a general overview of the pathogen as well as susceptibility patterns and resistance notes; this information was obtained from readily available reference resources and medical literature.^37–39^ Antimicrobial information includes dosing by age, general spectrum of activity, pharmacology, adverse effects, interactions, and drug monitoring; this was curated by our ASP teams and made consistent with institution-specific LEXI-COMP® information.

Several members of the ASP team reviewed and updated the content multiple times during the prerelease phase to optimize clinical accuracy and usability. The team, along with designated subject matter experts, reviewed all clinical guidelines prior to final approval to ensure accuracy and enhance navigation. During beta-testing, pharmacists, hospital medicine fellows and faculty, ID faculty, emergency medicine faculty, a human factors expert, and clinical effectiveness team members provided structured feedback for further revision prior to launch.

#### Firstline adoption

Adoption of Children’s Colorado or Denver Health Firstline guidelines among outside facilities was measured via REDCap user feedback surveys (Supplemental File 1).

### Implementation

#### Implementation of the Firstline app

Both mobile app versions launched in October 2023, followed by a multifaceted media campaign in November 2023. Children’s Colorado institutional implementation included (1) in-person advertisement during daily handshake ASP rounds,^12^ (2) adding the Colorado-specific Firstline link to the Children’s Colorado clinical pathways page,^30^ (3) installation of the app onto hospital-provided phones, (4) promotion through several hospitalwide clinical newsletters, (5) distribution to clinical teams via E-mail, and (6) promotion of Firstline on an institutionally sponsored podcast and webinar.^40,41^

At Denver Health, the Firstline app was not disseminated internally because a preexisting and widely utilized ASP app already served this purpose.^42,43^ As such, the creation of the Denver Health Firstline app was intended for utilization by Colorado practitioners external to Denver Health. Advertisement of both Children’s Colorado and Denver Health Firstline apps to external providers included dissemination to hospital ASP contacts through CDPHE, presentation of Firstline during local, regional, and national conferences, and distribution via regional care network and Colorado American Academy of Pediatrics newsletters.

#### Patterns of Firstline utilization in practice

The Firstline analytics dashboard compiled de-identified user engagement data, accessible in real-time by the evaluation team. Trackable data included the most frequently viewed content, further stratified by location, role, and practice setting, to assess how Firstline was utilized in practice.

### Maintenance

#### Sustained use over time

To assess the sustainability of the Children’s Colorado and Denver Health Firstline apps, we tracked the number of monthly average users (counted as engaging with Firstline once in a 28-day period) from launch through December 31^st^, 2025.

#### Ongoing content updates

Following launch, the app has undergone continuous iterative refinement, with weekly updates to improve usability and enhance clarity based on real-time user feedback. Clinical content has been expanded and revised to reflect evolving needs, including guidance in response to the recent measles outbreak.

#### Funding sustainability

Institutional commitment to ongoing funding was assessed as a marker of organizational-level maintenance.

### Data analysis

The number of Firstline users and usage characteristics was summarized using standard descriptive statistics. Bar charts displayed use patterns and user demographics. A line chart displayed the monthly number of Firstline users over time. Descriptive statistics summarized all survey items. Tables report the number and percentage of respondents selecting each response option. Calculations used the total number of respondents for each question as the denominator, excluding missing or skipped responses.

### Ethics

This study was reviewed by the Children’s Colorado Organizational Research Risk and Quality Improvement Review Panel and deemed as Quality Improvement, exempt from Human Subjects Research review.

## Results

### Reach

#### Firstline utilization and usage patterns

From launch through September 30^th^, 2024 (∼12 mo), there were a total of 4,506 mobile users among both platforms (3,619 for Children’s Colorado and 887 for Denver Health). Of these, 4,409 users were located within Colorado, comprising 15% of the 30,196 Colorado clinicians (Table 1). Most Firstline users (78%) were located within the Denver Metro region, where the majority of clinicians are located (n = 18,251), representing 19% of clinicians in the Denver Metro area (Table 1). In rural counties (non-Denver Metro), use varied from 5% to 17% of eligible clinicians (Table 1).

**Table 1.** Percent Firstline use by Colorado region. The number of Firstline users from launch through September 30th, 2024 represents the numerator, and clinicians (physicians, advanced practice providers, and pharmacists) per region in Colorado represents the denominator Table 1 long description.

Colorado region	Firstline users	Clinicians	% Firstline use by region
Denver Metro	3,448	18,251	**19**
Front Range	343	4,273	**8**
Northeast	37	216	**17**
Northwest	259	1,921	**13**
South Central	182	3,778	**5**
Southeast	41	864	**5**
Southwest	99	893	**11**
**Total**	**4,409**	**30,196**	**15**

Abbreviations: %, percent; Metro, metropolitan.

A total of 47,836 sessions occurred from launch through December 31^st^, 2025 (41,159 within Children’s Colorado and 6,677 within Denver Heath platforms). Similar to total use, the greatest number of sessions occurred within the Denver Metro region (83.1%; Table 2). Colorado’s rural counties (non-Denver Metro) accounted for 7,222 sessions (15.1%). Almost two percent of all sessions occurred in surrounding states outside of Colorado (Arizona, Idaho, Montana, Nevada, New Mexico, Utah, and Wyoming; Table 2).

**Table 2.** Number of sessions (an interaction within a 30-min window, by geocoded location) from launch through December 31st, 2025 in Children’s Colorado and Denver Health Firstline application by region Table 2 long description.

Region	Children’s Colorado sessions	Denver health sessions	Total sessions (%)
Colorado			
Denver Metro	35,678	4,064	39,742 (83.1)
Front range	2,142	406	2,548 (5.3)
Northeast	68	131	199 (.4)
Northwest	924	471	1,395 (2.9)
South Central	739	173	912 (1.9)
Southeast	127	105	232 (.5)
Southwest	773	1,163	1,936 (4)
Outside Colorado	708	164	872 (1.8)
**Total**	**41,159**	**6,677**	**47,836**

Abbreviations: Metro, metropolitan.

#### Demographics of Firstline users

**Figure 1.**
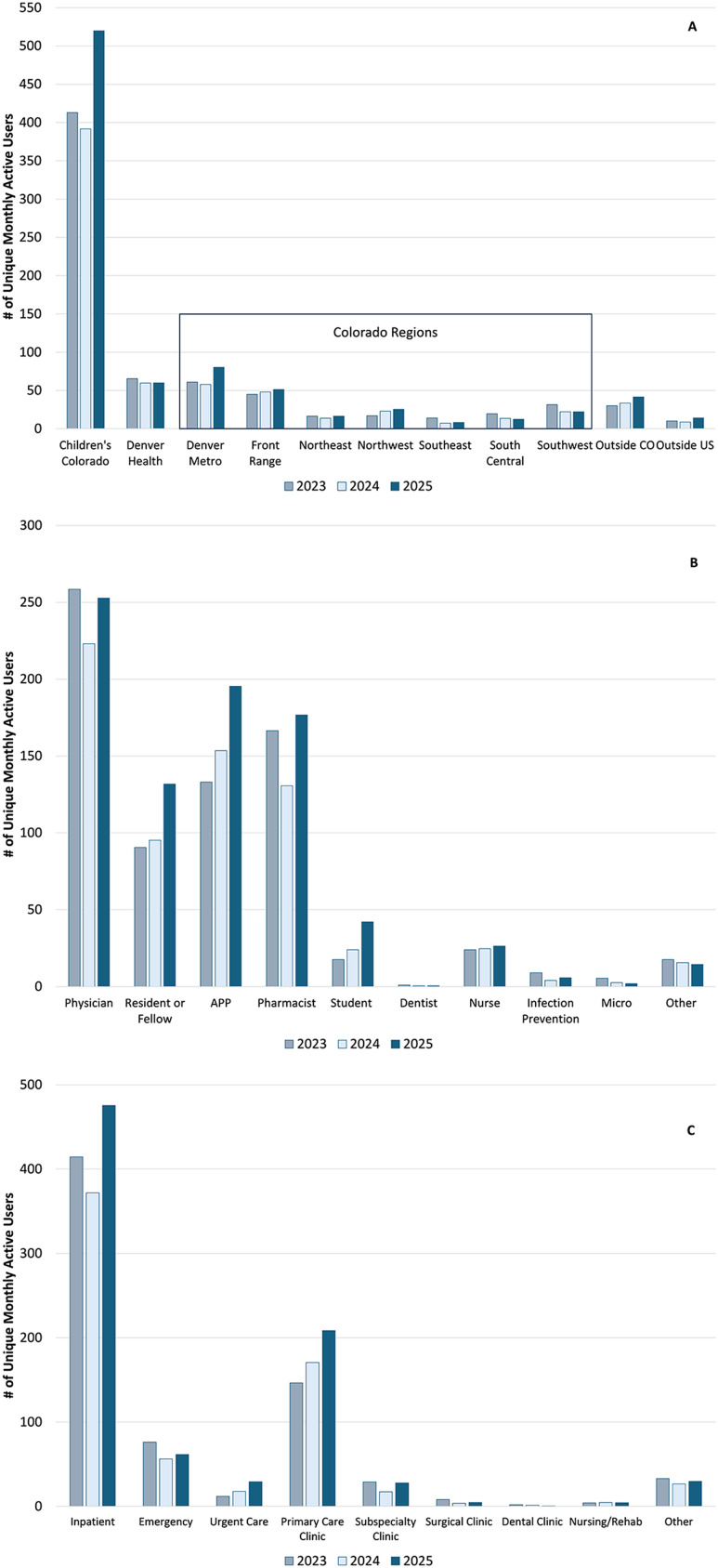
Bar graph displaying the average total monthly active mobile users among both platforms (Children’s Colorado and Denver Health) for 2023 to 2025 by location (1A), practitioner type (1B), and practice setting (1C). #, number; CO, Colorado; US, United States; APP, advanced practice provider; Micro, microbiologist; Rehab, rehabilitation center. Figure 1 long description.

Most Firstline users were located within Children’s Colorado network of care, with increasing use in 2025 within the Denver Metro, Front Range, and Northwest Colorado regions and outside Colorado (Figure 1A). Most frequent users were physicians, APPs, pharmacists, and residents/fellow physicians. Use among students and nursing providers was low but increased in 2025 (Figure 1B). Most users were located within the inpatient setting, followed by primary care clinics and the emergency department. In 2025, increased engagement in inpatient, primary care, and urgent care settings was noted (Figure 1C).

### Effectiveness

There were 108 responses on the initial Firstline user feedback survey (April 2024) and 107 responses on the follow-up survey (February 2025; Table 3). Children’s Colorado users contributed the most responses (75.9% and 66.4%; Table 3). The follow-up survey captured more responses from the Denver Metro area (increasing from 6.5% to 13.1%) and outside the Denver Metro area (from 10.2% to 15.9%; Table 3). Respondents included 34% physicians, 31% APPs, 16% pharmacists, and 14% residents or fellows (total from both surveys; data not shown). Among Denver Metro respondents, 90% worked in primary care settings, whereas for non-Denver Metro regions, 40% of respondents worked in inpatient settings and 43% in primary care (data not shown).

**Table 3.** Summary of Firstline user feedback survey responses from initial survey (April 2024) and follow-up survey (February 2025) Table 3 long description.

	Initial Survey n = 108		Follow-up Survey n = 107	
	#	%	#	%
**Location**				
Children’s Hospital Colorado NOC	82	75.9	71	66.4
Denver Health	6	5.6	0	0.0
Denver Metro CO	7	6.5	14	13.1
Front Range CO	4	3.7	5	4.7
Southwest CO	4	3.7	4	3.7
Southeast CO	0	0.0	2	1.9
South Central CO	0	0.0	1	0.9
Northwest CO	3	2.8	5	4.7
Outside CO	2	1.9	4	3.7
Outside US	0	0.0	1	0.9
**Use frequency**				
Daily	13	12.0	19	17.8
1–3 times a week	54	50.0	49	45.8
1–3 times a month	35	32.4	25	23.4
Less than once a month	6	5.6	14	13.1
**Ease of app**				
Very easy	69	63.9	78	72.9
Pretty easy	35	32.4	26	24.3
Somewhat easy	4	3.7	3	2.8
**Most Frequently Used Section (multi-selection)**				
Guidelines	72	28.5	76	29.8
Antimicrobials	92	36.4	87	34.1
Pathogens	65	25.7	63	24.7
Resources	24	9.5	29	11.4
**Enhanced Antibiotic Selection Knowledge**				
Very much	41	38.0	54	50.5
Quite a bit	36	33.3	35	32.7
Somewhat	22	20.4	16	15.0
Slightly	9	8.3	1	0.9
Not at all	0	0.0	1	0.9
**Enhanced Antibiotic Dosing Knowledge**				
Very much	43	39.8	59	55.1
Quite a bit	37	34.3	28	26.2
Somewhat	22	20.4	15	14.0
Slightly	5	4.6	4	3.7
Not at all	1	0.9	1	0.9
**Enhanced Guideline Utilization**				
Very much	40	37.0	54	50.5
Quite a bit	36	33.3	28	26.2
Somewhat	18	16.7	17	15.9
Slightly	7	6.5	5	4.7
Not at all	7	6.5	3	2.8
**Ongoing Firstline Support**				
Yes	108	100	107	100

N, total number; #, number; %, percent; NOC, network of care; CO, Colorado.

Overall, respondents reported using Firstline at least one to three times a week (50% and 45.8%), found Firstline very easy to use (63.9% and 72.9%), reported enhanced antibiotic selection knowledge (71.3% and 83.2%), antibiotic dosing knowledge (74.1% and 81.3%), and guideline utilization (70.4% and 76.6%; Table 3). Though responses were limited, non-Denver Metro respondents reported greater knowledge gains overall than Denver Metro respondents (data not shown). 100% of respondents in both surveys advocated for ongoing support for Firstline (Table 3).

### Adoption

Survey data revealed that 7% of all respondents reported having no internal guidelines at their facilities (Table 4); 38% of facilities with no guidelines were located in the Denver Metro region and 50% in the rural, non-Denver Metro regions (data not shown). Additionally, 10% of respondents reported that their facilities utilized guidelines adopted from Children’s Colorado or Denver Health Firstline app (Table 4). Of these, 57% were located in the Denver Metro region and 43% in the rural, non-Denver Metro regions (data not shown).

**Table 4.** Antimicrobial stewardship characteristics of Firstline users obtained from user feedback survey responses (initial survey April 2024, follow-up February 2025) Table 4 long description.

	Initial survey n = 108		Follow-up survey n = 107		Total n = 215	
	#	%	#	%	#	%
**ASP program**						
Yes	92	85	85	79	177	82
No	13	12	17	16	30	14
Unknown/Blank	3	3	5	5	8	4
**Facility Guideline Utilization**						
Facility-specific guidelines	93	86	80	75	173	80
Guidelines adopted from Firstline	7	6	14	13	21	10
Guidelines adopted from other source	1	1	2	2	3	1
No guidelines	7	6	9	8	16	7
Unknown/Blank	0	0	2	2	2	1
**Monitor Guideline Adherence**						
Yes	92	85	84	79	176	82
No	5	5	6	6	11	5
Unknown/Blank	11	10	17	16	28	13

N, total number; #, number; %, percent; ASP, antimicrobial stewardship program.

### Implementation

**Figure 2.**
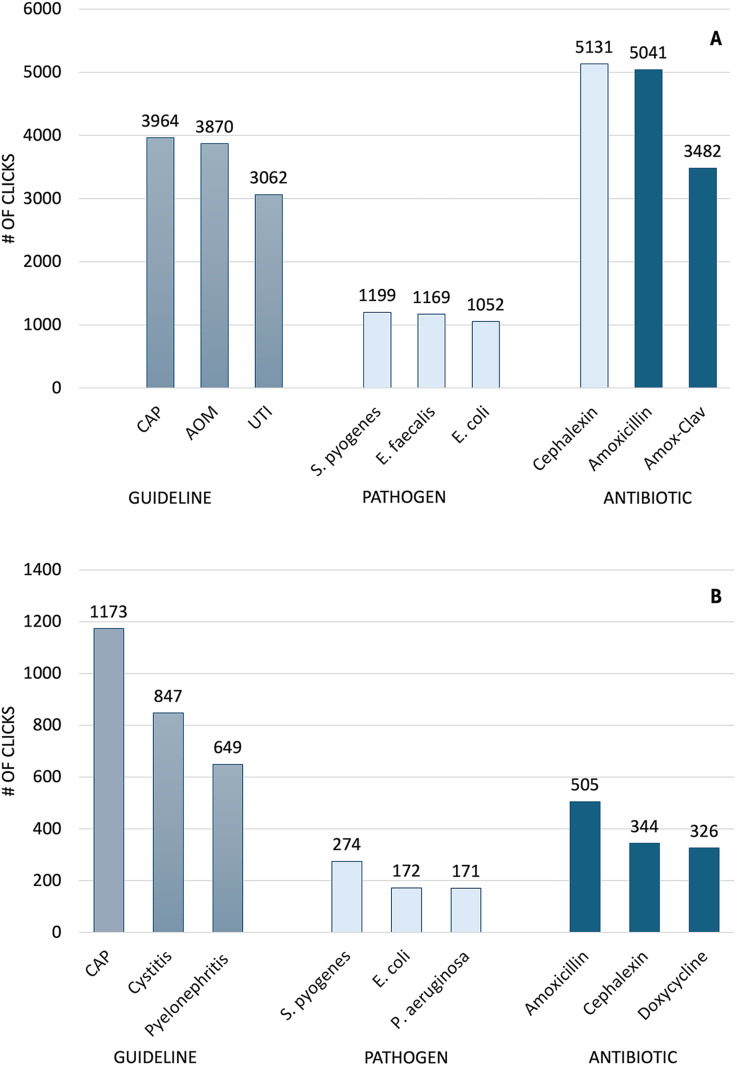
Bar graph displaying the most commonly utilized Firstline guidelines, pathogens, and antibiotics among users of the Children’s Hospital Colorado (Figure 2A) and Denver Health (Figure 2B) platforms. #, number; CAP, community acquired pneumonia; AOM, acute otitis media; UTI, urinary tract infection; amox-clav, amoxicillin-clavulanate. Figure 2 long description.

Users on both platforms (Children’s Colorado and Denver Health) most frequently accessed the guideline for uncomplicated community-acquired pneumonia and the pathogen *Streptococcus pyogenes* (Figure 2). Cephalexin was the most often viewed antimicrobial among Children’s Colorado users (Figure 2A), and amoxicillin was most frequently viewed among Denver Health users (Figure 2B).

### Maintenance

#### Sustained use over time

**Figure 3.**
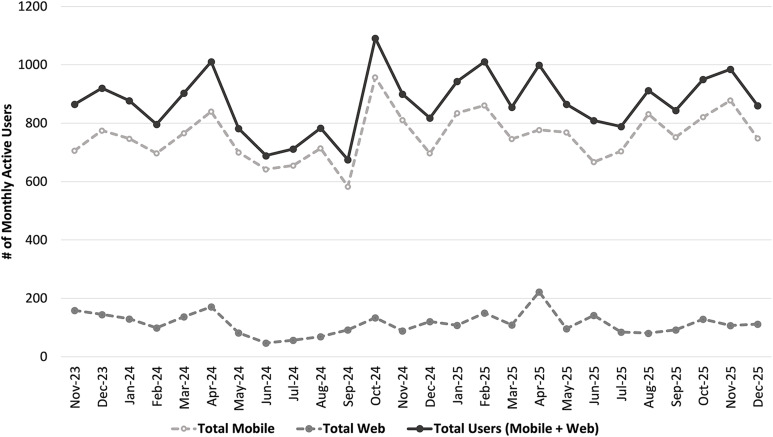
Line chart displaying the total monthly active users among both platforms (Children’s Colorado and Denver Health) from November 2023 through December 2025. The solid black line represents the total number of users (mobile and website), the dashed light gray line represents the total mobile users, and the dark gray dashed line represents the total website users. #, number. Figure 3 long description.

There was an average of 871 unique monthly users (757 average mobile users, 114 web) from launch through December 2025 among both platforms (Children’s Colorado and Denver Health) with consistent use over time (Figure 3).

#### Institutional commitment

In August 2025, Children’s Colorado leadership allocated ongoing funding to support ongoing use of the Firstline app.

## Discussion

In this pragmatic study, we present our experience developing and implementing a mobile ASP tool, Firstline, to address barriers to robust ASP throughout Colorado hospitals.^25,27,28^ Using the RE-AIM framework, we demonstrate increasing reach, high user-reported satisfaction, organizational adoption, and sustained engagement over time.

Implementation of the Firstline mobile app across Colorado was associated with an uptake of 4,409 downloads representing 15% of practitioners in the state within the first year. Though most of the use is within the Denver Metro region, increasing use in other regions (Front Range, Northeast, and Northwest Colorado) over time demonstrates rising incorporation into rural and frontier areas. The most frequently accessed content suggests that Firstline is meeting the needs of frontline clinicians for rapid access to common, high-impact ID information. Sustained engagement, as evidenced by an average of 871 unique monthly users over two years, supports ongoing integration of Firstline into routine clinical practice. User feedback surveys demonstrated high satisfaction and perceived impact on clinical practice. Notably, 100% of surveyed respondents advocated for ongoing Firstline support, underscoring the value of this resource. Though prescribing behavior was not directly measured, these findings align with prior literature showing that mobile applications can improve clinician knowledge and confidence in antimicrobial prescribing.^31–33^

Firstline is freely accessible to all users, which promotes equitable access to robust ASP resources, particularly in rural and critical access hospitals. Another strength of Firstline is the ability for hospitals to adopt or adapt content, particularly guidelines, to meet hospital ASP regulatory requirements. Creation of up-to-date institutional guidelines, particularly given many national guidelines are outdated/retired, is time consuming, and requires expertise that is not always available at rural or frontier hospitals. Our initial data shows that 10% of surveyed respondents reported institutional adoption of guidelines derived from the Children’s Colorado or Denver Health Firstline app, demonstrating organizational-level uptake beyond individual use and meeting hospital needs for clinical guidelines.

This project displays a unique initiative of two large academic institutions collaborating with public health to improve stewardship among all Colorado hospitals through the use of a mobile app. Various studies have evaluated using a mobile app as an ASP tool; however, the majority of these studies focus on a single institution or within a single health system.^31,33,34,43^ Similarly, many studies that have implemented ASP programs at smaller hospitals have focused on a single health system.^22,23^ Our work aligns with trends seen among several public health systems which are leveraging mobile technology to provide user-friendly guidance to standardize practice, support decision making, and cultivate collaborations.^44,45^

Limitations of this work include an inability to assess impact on antibiotic prescribing. While such databases are available, they are generally either very delayed, costly, or both. The location of use data is imprecise, while we can say that 17% of practitioners in the Northeast Colorado use Firstline, we cannot pinpoint that use to a particular hospital or clinic, limiting our knowledge of target facility uptake. To calculate Firstline use by region, both the numerator and denominator are imprecise. The numerator includes all practitioners (eg, nurses, students) in addition to physicians, APPs, and pharmacists, inflating our reach estimates; though physicians, APPS, and pharmacists comprise 89% of users. The denominator is also overestimated, as many on the list never or rarely prescribe antibiotics. Notably, pharmacists were included in the denominator as they represent a high number of Firstline users. Lastly, survey response rates were low compared to total users, and respondents may not be representative of all users.

With this “stewardship in your pocket” model, we describe a unique, scalable approach to extending ASP support beyond academic medical centers, meeting the identified needs of smaller hospitals. Future goals include correlating app use with antimicrobial prescribing to assess clinical impact, expanding reach to non-Denver metro regions and emergency and urgent care settings, and expanding ASP reach by engaging nursing staff through use of the app.

## Supporting information

10.1017/ash.2026.10418.sm001 Dimo et al. supplementary material Dimo et al. supplementary material

## Supplementary material

The supplementary material for this article can be found at https://doi.org/10.1017/ash.2026.10418.

## Research transparency and reproducibility

Department of Regulatory Agencies Professional and Occupational Licenses Information Marketplace: https://data.colorado.gov/Regulations/Professional-and-Occupational-Licenses-in-Colorado/7s5z-vewr/about_data.

## Table 1. Long description

The table presents data on Firstline users and clinicians across different regions in Colorado. It includes columns for the Colorado region, Firstline users, Clinicians, and percentage of Firstline use by region. The Denver Metro region has the highest number of Firstline users at 3,448 and 18,251 clinicians, representing 19 percent of Firstline use. The Front Range region has 343 Firstline users and 4,273 clinicians, with 8 percent Firstline use. The Northeast region has 37 Firstline users and 216 clinicians, with 17 percent Firstline use. The Northwest region has 259 Firstline users and 1,921 clinicians, with 13 percent Firstline use. The South Central region has 182 Firstline users and 3,778 clinicians, with 5 percent Firstline use. The Southeast region has 41 Firstline users and 864 clinicians, with 5 percent Firstline use. The Southwest region has 99 Firstline users and 893 clinicians, with 11 percent Firstline use. In total, there are 4,409 Firstline users and 30,196 clinicians across Colorado, with an overall Firstline use percentage of 15.

Navigate back to Table 1..

## Table 2. Long description

The table presents data on the number of sessions in Children’s Colorado and Denver Health Firstline application by region. It includes three columns: Region, Children’s Colorado sessions, Denver health sessions, and Total sessions percentage. The table has 9 rows, including a total row. The Denver Metro region has the highest number of sessions with 39,742 total sessions, accounting for 83.1 percentage of the total. The Front Range, Northeast, Northwest, South Central, Southeast, Southwest, and Outside Colorado regions have fewer sessions, with the Southwest region having 1,936 sessions and the Northeast region having the least with 199 sessions. The total number of sessions is 47,836, with 41,159 in Children’s Colorado and 6,677 in Denver Health.

Navigate back to Table 2..

## Figure 1. Long description

The image contains three bar graphs. The first graph (1A) displays the average total monthly active mobile users among both Children’s Colorado and Denver Health platforms by location for the years 2023 to 2025. The locations include Children’s Colorado, Denver Health, Denver Metro, Front Range, Northeast, Northwest, Southeast, South Central, Southwest, Outside Colorado, and Outside the United States. The second graph (1B) shows the average total monthly active mobile users by practitioner type, including Physician, Resident or Fellow, Advanced Practice Provider, Pharmacist, Student, Dentist, Nurse, Infection Prevention, Microbiologist, and Other. The third graph (1C) illustrates the average total monthly active mobile users by practice setting, such as Inpatient, Emergency, Urgent Care, Primary Care Clinic, Subspecialty Clinic, Surgical Clinic, Dental Clinic, Nursing/Rehabilitation, and Other. Each graph uses different shades of blue to represent the years 2023, 2024, and 2025. The values are estimated and show trends over the three-year period.

Navigate back to Figure 1..

## Table 3. Long description

The table presents data from two surveys: an initial survey with 108 responses and a follow-up survey with 107 responses. It compares various locations, use frequency, ease of app, most frequently used sections, enhanced antibiotic selection knowledge, enhanced antibiotic dosing knowledge, enhanced guideline utilization, and ongoing firstline support. Key locations include Children’s Hospital Colorado NOC, Denver Health, Denver Metro CO, Front Range CO, Southwest CO, Southeast CO, South Central CO, Northwest CO, Outside CO, and Outside US. The table shows percentages and counts for each category, highlighting trends and changes between the two surveys. For instance, Children’s Hospital Colorado NOC had the highest response rates in both surveys, while Denver Metro CO saw an increase in responses in the follow-up survey.

Navigate back to Table 3..

## Table 4. Long description

The table presents data on antimicrobial stewardship program characteristics based on user feedback surveys conducted in April 2024 and February 2025. It includes responses from 215 participants, with 108 in the initial survey and 107 in the follow-up survey. The table is divided into three main sections: ASP program, Facility Guideline Utilization, and Monitor Guideline Adherence. Each section provides the number and percentage of responses for each category. For the ASP program, 82 percent of total respondents reported having an ASP program, 14 percent reported not having one, and 4 percent were unknown or left blank. Facility-specific guidelines were utilized by 80 percent of respondents, while 10 percent adopted guidelines from Firstline, 1 percent from other sources, 7 percent had no guidelines, and 1 percent were unknown or left blank. For monitoring guideline adherence, 82 percent of respondents indicated they do monitor adherence, 5 percent do not, and 13 percent were unknown or left blank. The table highlights trends and comparisons between the initial and follow-up surveys, showing slight variations in responses over time.

Navigate back to Table 4..

## Figure 2. Long description

The bar graph compares the most commonly utilized Firstline guidelines, pathogens, and antibiotics among users of the Children’s Hospital Colorado and Denver Health platforms. The x-axis lists categories: guideline, pathogen, and antibiotic. The y-axis measures the number of clicks. The graph has two sections: A and B. Section A shows three guidelines: CAP with 3964 clicks, AOM with 3870 clicks, and UTI with 3062 clicks; three pathogens: S. pyogenes with 1199 clicks, E. faecalis with 1169 clicks, and E. coli with 1052 clicks; and three antibiotics: Cephalexin with 5131 clicks, Amoxicillin with 5041 clicks, and Amox-Clav with 3482 clicks. Section B shows three guidelines: CAP with 1173 clicks, Cystitis with 847 clicks, and Pyelonephritis with 649 clicks; three pathogens: S. pyogenes with 274 clicks, E. coli with 172 clicks, and P. aeruginosa with 171 clicks; and three antibiotics: Amoxicillin with 505 clicks, Cephalexin with 344 clicks, and Doxycycline with 326 clicks. All values are approximated.

Navigate back to Figure 2..

## Figure 3. Long description

A line chart displaying the total monthly active users among both platforms from November 2023 through December 2025. The solid black line represents the total number of users (mobile and website), the dashed light gray line represents the total mobile users, and the dark gray dashed line represents the total website users. The x-axis represents the months from November 2023 to December 2025, and the y-axis represents the number of monthly active users, ranging from 0 to 1200. The total number of users fluctuates between approximately 700 and 1100, with peaks around March 2024 and October 2024. The total mobile users range between approximately 600 and 900, while the total website users range between approximately 100 and 200. All values are approximated.

Navigate back to Figure 3..
