# Supplementary material for: Stewardship in your pocket: launching Colorado’s free antimicrobial mobile application
Source: Antimicrob Steward Healthc Epidemiol. 2026 Jun 1;6(1):e158. doi: 10.1017/ash.2026.10418 (PMC13227117; doi:10.1017/ash.2026.10418)
Supplement: Dimo et al. supplementary material [file S2732494X26104185sup001.pdf]

Children's Hosptial Colorado Firstline User Survey

Please help us help you! We'd appreciate a few minutes of your time with the following survey to give us feedback on the Children's Hospital Colorado Firstline mobile app. The Children's Colorado Firstline app is a free resource that is publicly available. This resource is currently funded by a grant through 2025. We hope to continue this resource beyond then, but need your feedback to improve and advocate for funding!

Thank you!

Demographics

Please select the region where you spend the majority of your time working:

- ◯ Children's Hospital Colorado Practice Site (Anschutz, Network of Care, Colorado Springs)
- ◯ Denver Health Practice Site
- ◯ Denver Metro area (not Children's Colorado or Denver Health)
- ◯ Front Range
- ◯ Northwest Colorado
- ◯ Northeast Colorado
- ◯ Southeast Colorado
- ◯ South Central Colorado
- ◯ Southwest Colorado
- ◯ Outside Colorado, in the United States
- ◯ Outside United States

Please select your primary practitioner type:

- ◯ Resident or Fellow Physician (MD, DO, MBBS)
- ◯ Physician (MD, DO, MBBS)
- ◯ Pharmacist (PharmD, RPH)
- ◯ Physician Assistant or Nurse Practitioner (all license types)
- ◯ Nurse
- ◯ Dentist
- ◯ Epidemiologist or Infection Preventionist
- ◯ Microbiologist
- ◯ Student (medical, nursing, pharmacy, etc.)
- ◯ Other

What other practitioner type?

____

Please select your primary practice setting:

- ◯ Inpatient
- ◯ Nursing/Rehabilitation Facility
- ◯ Long-term Acute Care Hospital
- ◯ Primary Care Clinic
- ◯ Subspecialty Medical Clinic
- ◯ Surgical Clinic
- ◯ Dental Clinic
- ◯ Emergency Department
- ◯ Urgent Care
- ◯ Other

What other practice setting?

____

Please select your primary patient population:

- ◯ Pediatric
- ◯ Adult
- ◯ Pediatric and adult (e.g., Family Medicine)
- ◯ Geriatric
- ◯ Other

What other patient population?

____

Does your facility have an active Antimicrobial Stewardship Program (ASP)?

- ◯ Yes
- ◯ No
- ◯ Don't know

Are you part of the ASP team?

- ◯ Yes
- ◯ No

Does your facility utilize disease/condition specific pathways or clinical guidelines?

- ◯ Yes, utilize facility created and approved pathways
- ◯ Yes, utilize pathways adopted from Firstline (Children's Hospital or Denver Health)
- ◯ Yes, utilize pathways adopted from other source
- ◯ No
- ◯ Don't know

Does your facility monitor adherence to disease/condition specific pathways or clinical guidelines?

- ◯ Yes
- ◯ No
- ◯ Don't know

Mobile Application Use

Which area(s) of the Children's Colorado Firstline mobile app do you use most frequently (select all that apply)?

- ☐ Guidelines
- ☐ Resources and Infection Prevention
- ☐ Pathogens
- ☐ Antimicrobials

How easy is it for you to use the Children's Colorado Firstline mobile app?

- ◯ Very easy
- ◯ Pretty easy
- ◯ Somewhat easy
- ◯ Slightly easy
- ◯ Not at all easy

How often do you use the Firstline mobile app in your practice?

- ◯ Daily
- ◯ At least 1-3 times a week
- ◯ At least 1-3 times a month
- ◯ Less than 1 time a month

For what reason(s) do you primarily use the Children's Colorado Firstline app (select all that apply)?

- ☐ Clinical decision making
- ☐ Support with antibiotic choice or dose
- ☐ Review of antibiotic prescribing for antibiotic stewardship
- ☐ Review resources for antimicrobial stewardship or infection control
- ☐ Other

For what other reason do you use the Firstline app?

____

Antibiotic Use

Has the Children's Colorado Firstline mobile app enhanced your knowledge of antibiotic dosing for pediatric patients?

- ◯ Very much
- ◯ Quite a bit
- ◯ Somewhat
- ◯ Slightly
- ◯ Not at all

Has the Children's Colorado Firstline mobile app enhanced your knowledge of antibiotic selection for pediatric infections?	◯ Very much<br>◯ Quite a bit<br>◯ Somewhat<br>◯ Slightly<br>◯ Not at all
Has the Children's Colorado Firstline mobile app improved your utilization of clinical guidelines?	◯ Very much<br>◯ Quite a bit<br>◯ Somewhat<br>◯ Slightly<br>◯ Not at all
Feedback	
Do you think it is important to continue to support Statewide access to the Children's Colorado Firstline app in future years?	◯ Yes<br>◯ Maybe<br>◯ No
Do you have suggestions for the app (e.g., more guidelines, more pathogens and antimicrobials, other information)?	______________________________
Do you have suggestions on how to further disseminate the app?	______________________________
